# Visualizing research on the prognosis of papillary thyroid cancer: a bibliometric analysis

**DOI:** 10.3389/fonc.2026.1657719

**Published:** 2026-03-27

**Authors:** Pei Wang, Ming-Jun Wang, Qiu-Fen Mao, Sheng-Bo Han, Wen Liu, Chao-Qun Zhao, Cong Zhou

**Affiliations:** 1 Department of Radiation Oncology, Cancer Institute, The First Affiliated Hospital, and College of Clinical Medicine of Henan University of Science and Technology, Luoyang, China; 2 Department of Head and Neck Surgical Oncology, Cancer Institute, The First Affiliated Hospital, and College of Clinical Medicine of Henan University of Science and Technology, Luoyang, China; 3 Department of Nuclear Medicine, The First Affiliated Hospital, and College of Clinical Medicine of Henan University of Science and Technology, Luoyang, China; 4 Jining Medical University Clinical College, Affiliated Hospital of Jining Medical University, Jining, China; 5 Department of Thyroid Surgery, Clinical Research Center for Thyroid Disease of Yunnan Province, The First Affiliated Hospital of Kunming Medical University, Kunming, China; 6 Tianjin Dongli District Centers for Disease Control and Prevention, Tianjin, China; 7 School of Mental Health, Jining Medical University, Jining, China; 8 Department of Psychology, Affiliated Hospital of Jining Medical University, Jining, China; 9 Center for Evidence-Based Medicine, Jining Medical University, Jining, China

**Keywords:** bibliometric analysis, BRAF V600E, hotspots, papillary thyroid cancer, prognosis

## Abstract

**Background:**

Over the last two decades, the prognosis of papillary thyroid cancer (PTC) has attracted increasing research attention, highlighting its vital role in improving patient outcomes. However, despite the multitude of studies, comprehensive bibliometric analyses concentrating specifically on PTC prognosis are still scarce.

**Methods:**

Our study involved a bibliometric analysis of 3,430 articles related to PTC prognosis, derived from the Web of Science Core Collection (WOSCC) database. We utilized VOSviewer, CiteSpace, and the R package “bibliometrix” to examine publication trends, identify key contributing countries and institutions, map collaborative networks, recognize prominent journals, and scrutinize both high-frequency keywords and highly cited references.

**Results:**

From 2004 to 2024, the number of research articles on the prognosis of PTC steadily increased, with a total of 3,430 articles included in our analysis. The year 2022 marked the peak in the number of publications, with 325 articles being published. China and the United States are at the forefront in terms of publication volume and citations, albeit with a slightly lower citation rate for China. Notable contributions also emerge from South Korea, Italy, and Japan. Collaboration is predominantly observed among the leading nations, with developing countries engaging less frequently. Prominent institutions such as Kuma Hospital in Japan and Shanghai Jiao Tong University in China stand out in terms of publication output. The journals “Thyroid,” “Frontiers in Endocrinology,” and “World Journal of Surgery” lead the field, with “Thyroid” boasting the highest co-citation rate. The keyword analysis revealed six primary research clusters, focusing on cell and molecular biology, oncology-related terminology, risk factors, disease progression, and clinical treatment approaches. The highly cited articles underscore the significant impact of BRAF V600E mutations on PTC prognosis.

**Conclusion:**

This study offers an in-depth overview of prevailing research hotspots and trends, providing pivotal insights to direct future research endeavors and enhance prognostic care for PTC patients.

## 1 Introduction

Papillary thyroid cancer (PTC) is the most prevalent form of thyroid cancer, and the incidence of PTC is increasing in several countries (1). Classic PTC exhibits papillary structures and invasive nuclear features, with rare mitotic figures and common psammoma bodies, which are typically located in lymphatic vessels or stroma (2, 3). Patients with PTC may present with enlarged cervical lymph nodes. As the neck mass enlarges, it can compress and displace the trachea and esophagus, causing deformation. The high incidence and pronounced symptoms of PTC contribute to its increasing global burden. Nearly half of the global PTC burden is concentrated in South and East Asia. The burden of PTC in women is significantly greater than that in men and is also increasing annually (4, 5).

Treatment options for PTC are diverse and can be broadly categorized into surgical and conservative approaches. Surgical treatment can be further divided into total thyroidectomy and more conservative thyroid lobectomy. In general, except for patients meeting the criteria for papillary thyroid microcarcinoma, most PTC patients are recommended for total thyroidectomy. This procedure can thoroughly remove the tumor, reduce the risk of recurrence, and decrease the need for reoperation. During surgery, prophylactic central compartment lymph node dissection is performed to minimize the degree of lymphatic spread of cancer cells. Studies indicate that for tumors larger than 1 cm, the rate of local lymph node metastasis increases from 44.68% to 77.53% with increasing tumor size (6). These findings suggest that total thyroidectomy combined with central lymph node dissection is an aggressive and thorough treatment option, at least in the initial treatment stage. For patients with tumors smaller than 1 cm, without extrathyroidal extension, and no clinically apparent lymph node metastasis, thyroid lobectomy can be considered. Additionally, conservative treatments for PTC include radiotherapy, chemotherapy, thermal ablation, radioactive iodine-131 therapy, and targeted drug therapies. While the overall prognosis for PTC patients is generally favorable, it varies significantly with the size of the primary tumor. Tumors confined to the thyroid, smaller than 1 cm, or with micrometastases have a good prognosis. Conversely, distant metastasis and high invasiveness are associated with a poor prognosis (4, 5).

Over the years, the guidelines for the treatment of thyroid cancer, particularly those from the American Thyroid Association (ATA) and the European Thyroid Association (ETA), have evolved significantly. A notable shift has been observed toward less aggressive surgical approaches, especially for low-risk differentiated thyroid cancers (DTC). For instance, the ATA guidelines have increasingly emphasized the importance of individualized treatment plans on the basis of risk stratification, suggesting the need for less extensive surgery in certain low-risk patients (7, 8). Similarly, the ETA guidelines also recommend more conservative surgical options for patients with small tumors and no evidence of extrathyroidal extension (9). These revisions reflect a growing understanding of the heterogeneity of thyroid cancers and the need for tailored treatment strategies to optimize outcomes while minimizing morbidity.

Our study focused on the prognosis of PTC patients to provide insights for future treatment strategies. Bibliometrics is a method that quantitatively analyzes the information contained in the literature using statistical and mathematical techniques to objectively assess research outcomes, identify research hotspots, and forecast emerging trends (10). Currently, bibliometric analysis is widely applied in thyroid disease research. Wang et al. systematically summarized the status and future prospects of thyroid cancer immunotherapy by analyzing relevant literature in the Web of Science database (11). Song et al. supplemented research on pediatric thyroid cancer via bibliometric methods (12). Chen et al. conducted a bibliometric analysis of maternal hypothyroidism (13). However, very few bibliometric studies have focused on the prognosis of PTC. Consequently, we aimed to conduct a bibliometric analysis to assess the developmental framework, present state, and future directions in the research area of PTC prognosis. Our goal was to elucidate the evolving trends and collaborative dynamics in PTC prognosis research, offering valuable insights for future investigations and clinical practices.

## 2 Materials and methods

### 2.1 Data source and search strategy

**Figure 1:**
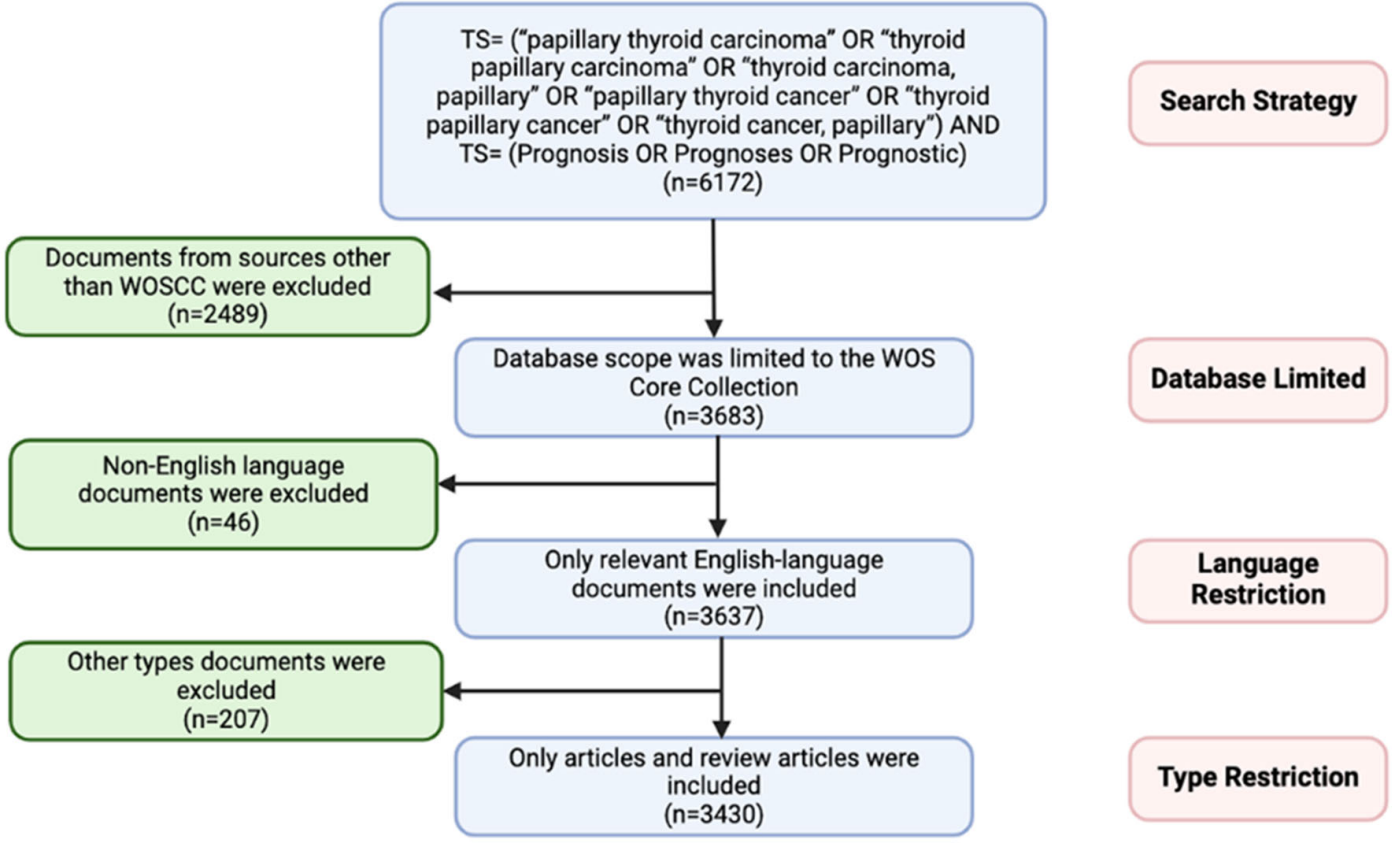
Flow chart illustrating the process of publication acquisition, filtering, and de-duplication for the bibliometric analysis of papillary thyroid cancer (PTC) prognosis.

In this study, all literature data were obtained from the Web of Science Core Collection database. This database encompasses academic publications from over 250 diverse global disciplinary fields and stands as a widely utilized platform for bibliometric analysis among research scholars (14–16). The bibliometric analysis in this article covers the period from January 1, 2004, to April 9, 2024. The search query employed was as follows: TS= (“papillary thyroid carcinoma” OR “thyroid papillary carcinoma” OR “thyroid carcinoma, papillary” OR “papillary thyroid cancer” OR “thyroid papillary cancer” OR “thyroid cancer, papillary”) AND TS= (Prognosis OR Prognoses OR Prognostic). Only English-language publications from the search results were included, and after excluding literature types such as letters, comments, and conference papers, a total of 3430 relevant articles were obtained. The detailed literature screening process is shown in **Figure 1**.

### 2.2 Data analysis

The study utilized various analysis tools, including Microsoft Office Excel 2021, VOSviewer (version 1.6.18), CiteSpace (version 6.1.R6), and the R package “bibliometrix,” for data analysis and visualization. Prior to analysis, we manually performed data cleaning and disambiguation to merge synonymous nodes and standardize author names and institutional affiliations (e.g., merging different spellings of the same institution, such as “Shanghai Jiao Tong University” and “Shanghai Jiao Tong Univ”) to ensure the accuracy of frequency counts and network linkages. Initially, data related to 1108 articles on papillary thyroid cancer (PTC) prognosis were uniformly extracted from the Web of Science Core Collection database, comprising information such as titles, authors, keywords, affiliations, countries/regions, citations, journals, and publication dates. This dataset underwent preliminary screening and processing before being analyzed via VOSviewer, CiteSpace, and the R package “bibliometrix” for bibliometric analysis. CiteSpace, known for creating knowledge maps in specific fields, was utilized for co-occurrence and clustering analysis of authors, research institutions, and countries (17, 18). VOSviewer, a bibliometric analysis software, facilitates the extraction and analysis of key information from publications, including collaboration relationships among countries, authors, institutions, and co-occurrence patterns of keywords (19, 20). For author and institution citation counts presented in **Tables 1**, **2**, self-citations were not excluded, as retaining them provides a more comprehensive reflection of overall academic influence within the field, consistent with common practice in bibliometric analysis. Finally, Bibliometrix, an open-source R package, was employed for comprehensive bibliometric and scientometric analysis, particularly for analyzing evolving trends of keywords in the literature (21).

**Table 1:** Ranking of the top ten major authors of PTC prognosis from 2004 to 2024.

Rank	Author	Documents	Total link strength	Countries/regions	institution	Author	Co- citations	Total link strength	Countries/regions	institution
1	Miyauchi, Akira	81	436	Japan	Kuma Hospital	Ito, Y	1839	18996	Japan	Kuma Hospital
2	Ito, Yasuhiro	74	426	Japan	Kuma Hospital	Xing, Mz	1333	16686	USA	Johns Hopkins University
3	Miya, Akihiro	63	408	Japan	Kuma Hospital	Haugen, Br	943	7836	USA	University of Colorado
4	Kihara, Minoru	42	288	Japan	Waseda University	Mazzaferri, El	883	10702	USA	The Ohio State University
5	Kobayashi, Kaoru	42	277	Japan	Chiba University	Hay, Id	750	8910	USA	Mayo Clinic
6	Takamura, Yuuki	38	281	Japan	Kuma Hospital	Cooper, Ds	597	6498	USA	Johns Hopkins University
7	Zhang, Hao	37	126	China	Jiangnan University	Nikiforov, Ye	519	7437	USA	University of Pittsburgh
8	Higashiyama, Takuya	35	259	Japan	Kuma Hospital	Davies, L	516	5883	USA	Manchester Metropolitan University
9	Ghossein, Ronald	30	45	USA	Memorial Sloan Kettering Cancer Center	Nikiforova, Mn	442	7200	USA	University of Pittsburgh
10	Tuttle, R. Michael	29	70	USA	Memorial Sloan Kettering Cancer Center	Jemal, A	388	2736	USA	American Cancer Society

**Table 2:** Ranking of the top ten major institutions of PTC prognosis from 2004 to 2024.

Rank	Institution	Publications	Original country	Institution	Total link strength	Original country	Institution	Citations	Original country
1	Kuma Hospital	85	Japan	University of Pisa	89	Italy	Johns Hopkins University	5508	USA
2	Yonsei University	84	South Korea	Johns Hopkins University	89	USA	Memorial Sloan Kettering Cancer Center	4912	USA
3	Shanghai Jiao Tong University	78	China	Memorial Sloan Kettering Cancer Center	72	USA	Kuma Hospital	4645	Japan
4	China Medical University	73	China	Seoul National University	69	South Korea	University of Pisa	3748	Italy
5	Sungkyunkwan University	72	South Korea	University of Pittsburgh	68	USA	University of Ulsan	2738	South Korea
6	Zhejiang University	72	China	University of Milan	65	Italy	The University of Sydney	2302	Australia
7	Memorial Sloan Kettering Cancer Center	64	USA	University of Padua	64	Italy	University of Pittsburgh	2214	USA
8	Fudan University	62	China	University of Perugia	61	Italy	University of Turin	2204	Italy
9	Sichuan University	59	China	The University of Sydney	59	Australia	University of Milan	2149	Italy
10	University of Ulsan	57	South Korea	Zhejiang University	48	China	University of Padua	2051	Italy

## 3 Results

### 3.1 Publication and citation analysis

**Figure 2:**
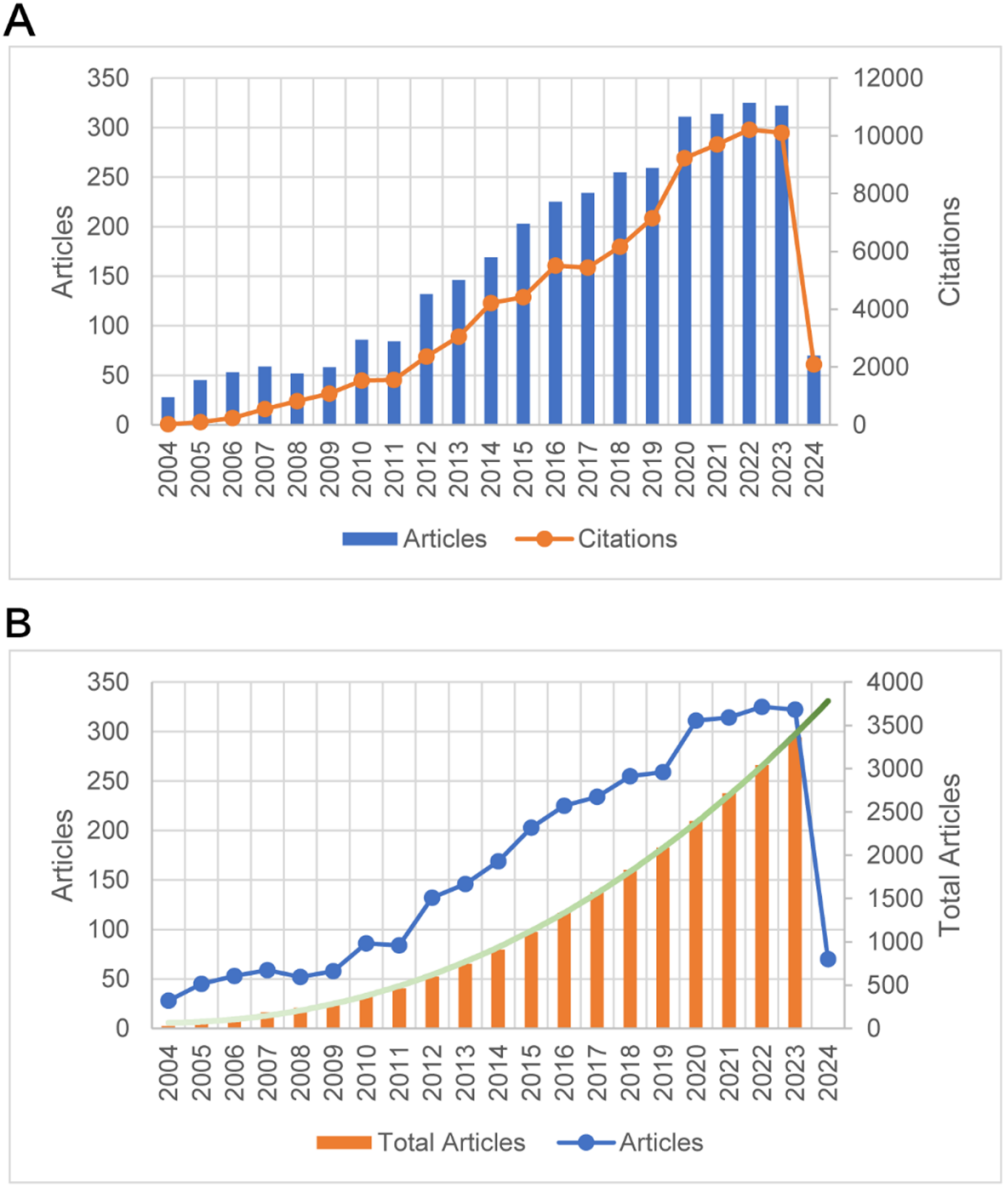
The analysis of annual publication quantity and citation frequency in the field of PTC prognosis. **(A)** The annual publication quantity and citation frequency of research on PTC prognosis from 2004 to 2024. **(B)** The annual publication quantity, cumulative publication quantity, and their polynomial fitting curves for PTC prognosis from 2004 to 2024.

**Figure 2A** illustrates the upward trends in both publication and citation counts from 2004 to 2024. Both metrics consistently increased during this period. Significant increases in publications were observed in 2010, 2012, and 2020, with a peak of 325 publications per year in 2022. Similarly, citation counts experienced notable growth during 2011–2014 and 2017-2020, likely attributed to influential publications preceding these periods. The highest citation peak, reaching 10,219 citations, was also recorded in 2022.

Furthermore, a polynomial fit was applied to the cumulative annual publication count, as depicted in **Figures 2B**, with the fitting equation y=0.0774x^3^+7.2983x^2^-10.613x+68.36 and a fitting goodness of R² = 0.9996.

### 3.2 Countries/regions analysis

Analysis of countries/regions contributing to the PTC prognosis literature offers insights into the global distribution of research outcomes and focus areas (**Table 3**). China and the United States have emerged as key players in this field, leading in both article production and citation rates. While China has a significantly higher number of articles (1279) than other countries, the United States surpasses in total citation frequency (26,954), indicating potentially greater influence. Notable contributions are also observed from countries such as South Korea, Italy, and Japan, further enriching the research landscape in PTC prognosis.

**Table 3:** Ranking of the top ten major countries/regions of PTC prognosis from 2004 to 2024.

Rank	Countries	Documents	Countries	Total link strength	Countries	Citations	Countries	H-index
1	China	1279	USA	297	USA	26954	Spain	40
2	USA	544	Italy	190	China	17285	Australia	38
3	South Korea	440	China	140	Italy	13501	Italy	34
4	Italy	266	Spain	85	South Korea	11941	Sweden	33
5	Japan	211	Australia	80	Japan	9076	USA	33
6	Turkey	109	Canada	78	Spain	4137	Canada	31
7	Brazil	93	Germany	65	Australia	3921	France	29
8	Germany	70	Japan	64	Canada	2813	Japan	29
9	Australia	69	Poland	63	Poland	2557	Poland	26
10	Poland	65	Czech Republic	60	Germany	2530	England	24

**Figure 3:**
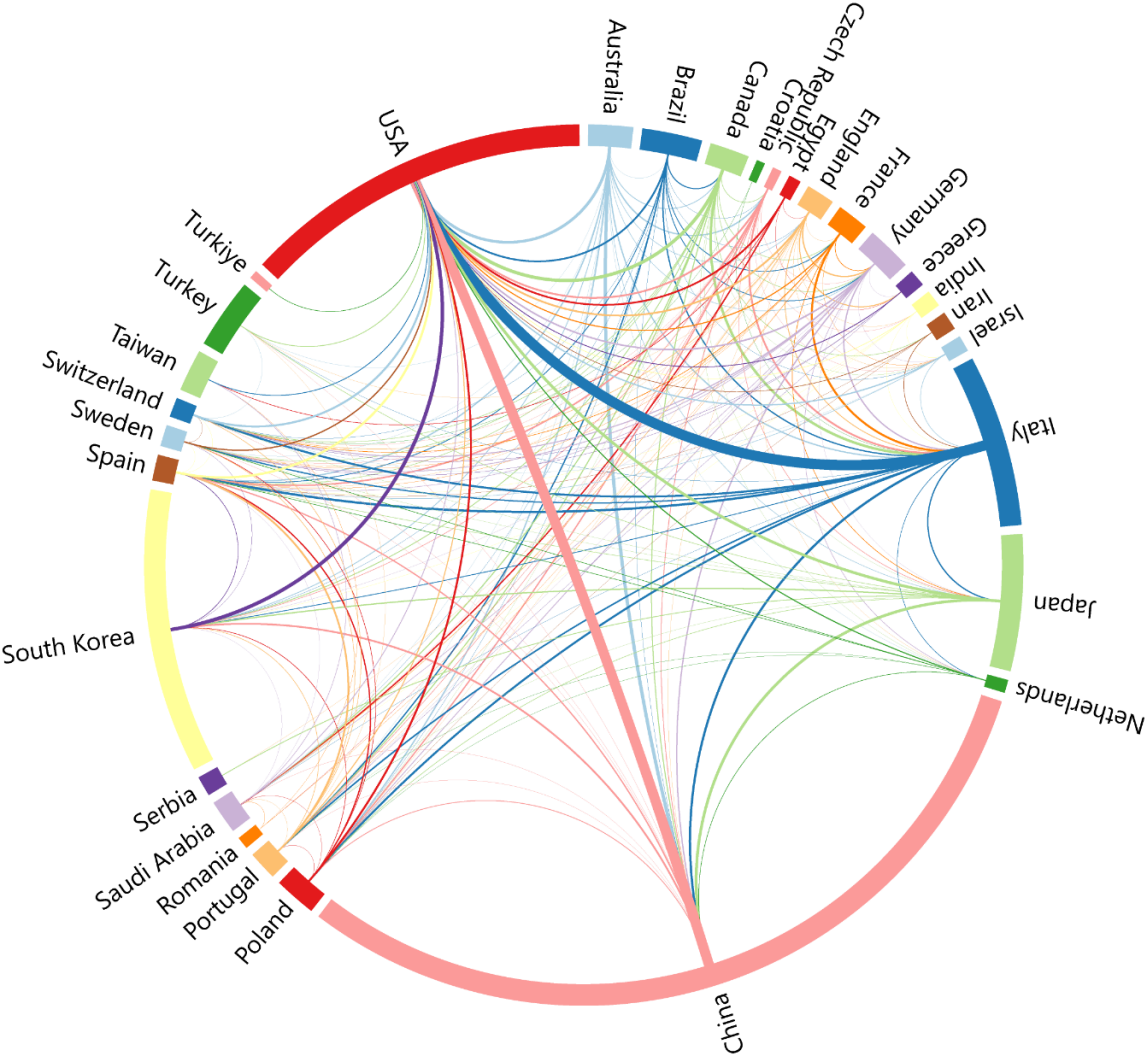
National collaborative network mapping of PTC prognosis from 2004 to 2024.

Using VOSviewer, we performed an extensive analysis of countries/regions. The collaboration dynamics among these nations are depicted in **Figure 3** via a chord diagram, with each country represented by differently colored ribbons reflecting collaboration strength. The findings highlight China as a leader in global collaboration, closely followed by the United States. Strong collaboration is observed between these two nations and between the United States and Italy. South Korea also plays a significant role in fostering academic exchanges, particularly with the United States.

### 3.3 Author analysis

**Table 1** presents the top ten authors on the basis of publication count and total co-citations. Leading contributors such as Miyauchi, Akira, Ito, Yasuhiro, Miya, and Akihiro have each authored over 50 publications in the field, notably all affiliated with Kuma Hospital in Japan. Regional diversity is evident among the top authors by publication count, with representations from South Korea, Japan, the USA, and China, which is consistent with the analysis of research dominance among countries/regions. Notably, the majority of authors with the highest total co-citations are from the USA, with only one exception from South Korea. Authors from institutions such as Johns Hopkins University and the University of Pittsburgh stand out among the top contributors.

**Figure 4:**
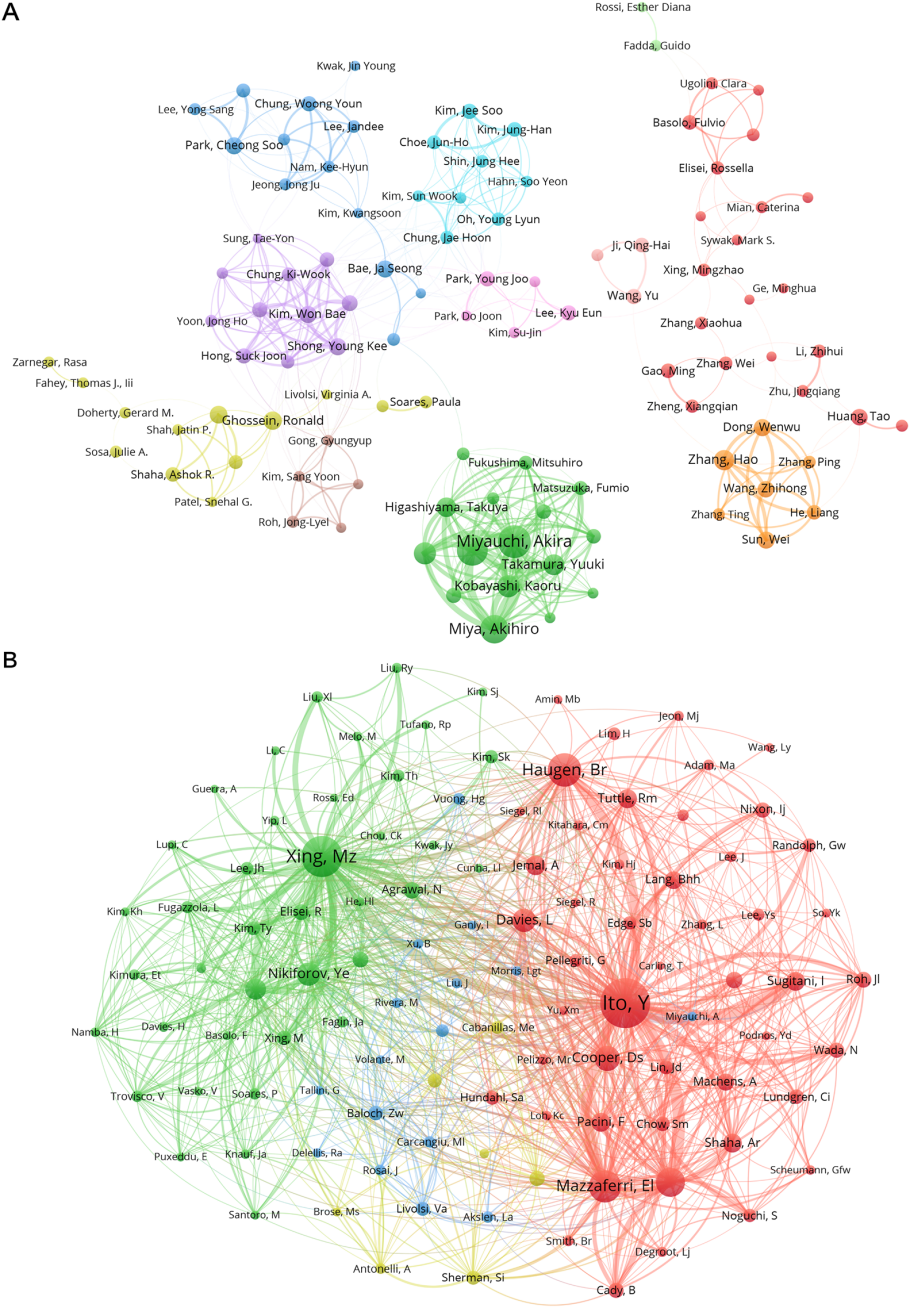
Author collaborative and citation network mapping of PTC prognosis from 2004 to 2024. **(A)** The map visualizes co-occurring authors in PTC prognosis research, with nodes of different colors reflecting authors in distinct clusters. Node size signifies the frequency of co-occurrence, and the links represent the co-occurrence relationships among authors. **(B)** The map visualizes co-cited authors in PTC prognosis research, where the node size corresponds to the frequency of their citations. This information is presented using VOSViewer to succinctly capture and analyze the interconnectedness of cited authors in the PTC prognosis research landscape.

To further illustrate the connections among these authors and their potential research similarities, the results of the analysis using VOSviewer are presented in **Figure 4**. In **Figures 4A**, different colors distinguish several major clusters, visualizing the collaboration relationships between authors within the same cluster. In **Figures 4A**, the largest author collaboration cluster, represented in green, centers around authors from Kuma Hospital in Japan, such as Miyauchi, Akira and Miya, Akihiro. Another significant cluster, shown in purple on the left, mainly includes authors from the University of Ulsan in South Korea, such as Chung, Ki-Wook and Kim, Won Bae. Various clusters of South Korean authors are visible, indicating geographical proximity and interconnectivity among them. Another notable cluster, highlighted in orange, primarily consists of Chinese authors, whereas the yellow cluster represents collaboration among American authors. Geographical proximity plays a role in author collaboration, but international collaboration is also significant, as seen in the red cluster with multinational collaboration, including authors from Italy, the USA, and Australia. Overall, the distribution of author collaboration clusters reveals spatial patterns, with most collaborations occurring among authors from the same country or institution, resulting in relatively independent clusters with minimal connections between them.

**Figure 4B** provides a visual representation of co-citation relationships between authors, indicating how frequently two authors are cited together by third-party authors in the same publication. This indirect co-citation relationship offers insights into potential connections and research relevance between these authors. While authors in this field cover a broad range of research topics, including neuroscience and the respiratory system, there are also more focused research directions within this broader scope. The authors are mainly divided into four clusters distinguished by color. In the red cluster on the right, the emphasis is primarily on clinical and surgical aspects. Authors such as Ito, Yasuhiro, Haugen, Br, Mazzaferri, El, and Davies, L have made significant contributions in this direction. The green cluster on the left predominantly focuses on cellular and molecular biology, featuring authors such as Xing, Mingzhao, Nikiforov, Ye, and Elisei, Rossella. Authors in the blue cluster in the middle primarily engage in pathology, laboratory medicine, or experimental medicine, including Baloch, Zubair W., Rosai, Juan, and Livolsi, V. A. The yellow cluster at the bottom encompasses authors with more diverse research directions, yet their studies also exhibit common themes in endocrinology, metabolism, and oncology.

### 3.4 Institution analysis

**Table 2** presents the top ten institutions in terms of the number of publications, total link strength, and total citations. Kuma Hospital in Japan has emerged as the most prolific institution in output in the field of PTC prognosis, with 85 publications, and it also ranks among the top three in terms of total citations (4645). The second most common institution is Yonsei University in South Korea, with 84 publications, while three institutions from China—Shanghai Jiao Tong University (78 publications), China Medical University (73 publications), and Zhejiang University (72 publications)—also make significant contributions to PTC prognosis research. Notably, Johns Hopkins University in the USA stands out as the institution with the highest number of total citations (5508), followed by Memorial Sloan Kettering Cancer Center (4912).

**Figure 5:**
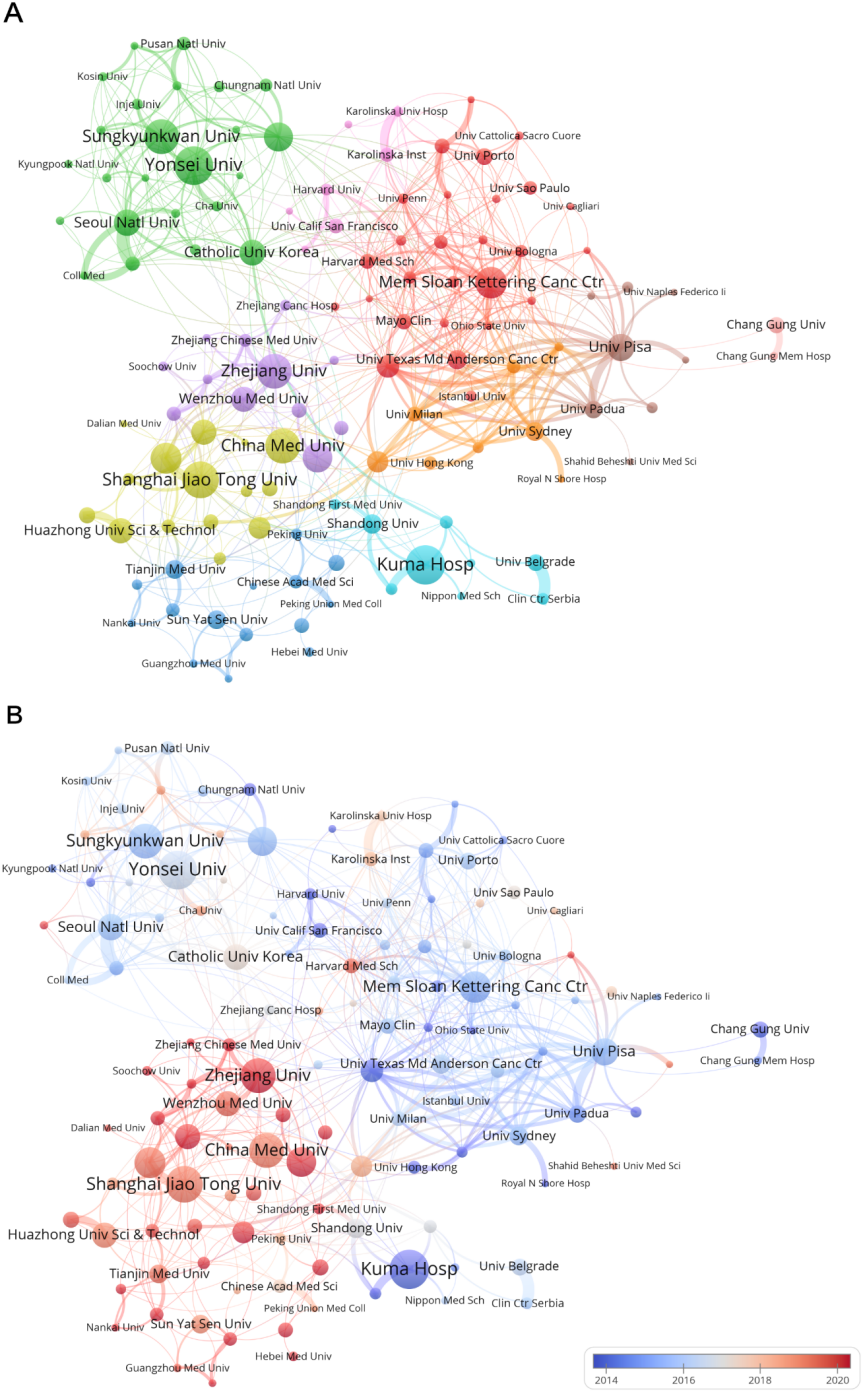
Institutional collaborative network mapping of PTC prognosis from 2004 to 2024. **(A)** The co-occurrence graph of research institutions is displayed, where node size indicates the frequency of co-occurrence, and connections represent the co-occurrence relationships. The size of each node reflects how often research institutions appear together, while the links signify the instances of their collaborative occurrences. **(B)** The figure depicts institutions’ recent contributions to PTC prognosis research relative to their overall output from 2004 to 2024, with a red bias indicating increased influence and a blue bias suggesting decreased activity in the field. The color scale reflects the ratio of keywords over the past five years, highlighting institutions with notable impacts or reduced involvement in this study.

**Figure 5** provides a visual representation of collaborative relationships among institutions, illustrating the spatial distribution of these collaborations. In **Figures 5A**, the upper left green cluster primarily gathers South Korean institutions with close collaborative ties, including several institutions that have made significant contributions to the field, such as Sungkyunkwan University, Seoul National University, and Yonsei University. The pink and red clusters on the right exhibit greater diversity in regions, predominantly featuring institutions from Europe and the USA, including Karolinska University Hospital in Sweden and the University of Porto in Portugal, as well as renowned American universities or medical centers such as Harvard Medical School, Memorial Sloan Kettering Cancer Center, and University of Texas MD Anderson Cancer Center. Although the brown cluster comprises fewer institutions, the University of Pisa, which has the strongest total link strength, is included in this cluster. Additionally, other Italian institutions such as the University of Naples Federico II and University of Padua are also present. The purple, yellow, and dark blue clusters at the bottom represent institutions in China, including influential institutions such as Shanghai Jiao Tong University, Zhejiang University, Huazhong University of Science and Technology, and Sun Yat-sen University. While the light blue and orange clusters also include Chinese institutions, they mainly depict multinational collaborations, such as academic exchanges between Kuma Hospital in Japan and Shandong University, and between the University of Sydney and the University of Milan.

**Figures 5B**, which extends the spatial analysis in **Figures 5A**, introduces a temporal dimension by examining the frequency of collaboration over the past five years. The color scheme highlights the chronological sequence of collaboration relationships on the basis of their occurrence frequency. This reveals that in the early years of this century, research on PTC prognosis was primarily conducted by institutions from Japan, South Korea, Europe, and the USA, with establishments such as Kuma Hospital and the University of Texas MD Anderson Cancer Center laying a solid foundation for the future development of this field. However, in recent years, there has been a noticeable shift, with academic collaboration among institutions in China injecting new vitality into this field.

### 3.5 Journal analysis

**Table 4** presents the top ten journals in terms of the number of publications and citations in this field. The journal with the highest number of publications is *Thyroid* (173 articles), which also has the highest citation count (9121 citations). Following *Thyroid*, we have *Frontiers in Endocrinology* (92 articles), *World Journal of Surgery* (85 articles), and *Cancers* (83 articles). The journals with the second highest citation count are *Journal of Clinical Endocrinology and Metabolism* (7532 citations) and *World Journal of Surgery* (3815 citations), indicating their central role in this field. More than half of the journals listed in the top rankings for both publication and citation frequency are high-impact journals in the Q1 or Q2 category. Given that the impact factor is an important indicator of journal quality, it can be inferred that articles on PTC prognosis published in these journals maintain a high level of quality.

**Table 4:** Ranking of the top ten major journals of PTC prognosis from 2004 to 2024.

Rank	Journal	Publications	IF(JCR2022)	JCR quartile	Co-Cited-Journal	Citations	IF(JCR2022)
1	Thyroid	173	6.6	Q1	Thyroid	9121	6.6
2	Frontiers in Endocrinology	92	5.2	Q1	Journal of Clinical Endocrinology and Metabolism	7532	5.8
3	World Journal of Surgery	85	2.6	Q2	World Journal of Surgery	3815	2.6
4	Cancers	83	5.2	Q2	Surgery	3656	3.8
5	Journal of Clinical Endocrinology and Metabolism	75	5.8	Q1	Cancer	2408	6.2
6	Annals of Surgical Oncology	66	3.7	Q2	Annals of Surgical Oncology	2403	3.7
7	Frontiers in Oncology	63	4.7	Q2	Cancer Research	2198	4.6
8	Medicine	57	1.6	Q3	Endocr-Relat Cancer	2073	3.9
9	Endocrine	54	3.7	Q3	Clinical Endocrinology	1749	3.2
10	Endocrine Journal	53	2	Q4	Oncogene	1516	8

**Figure 6:**
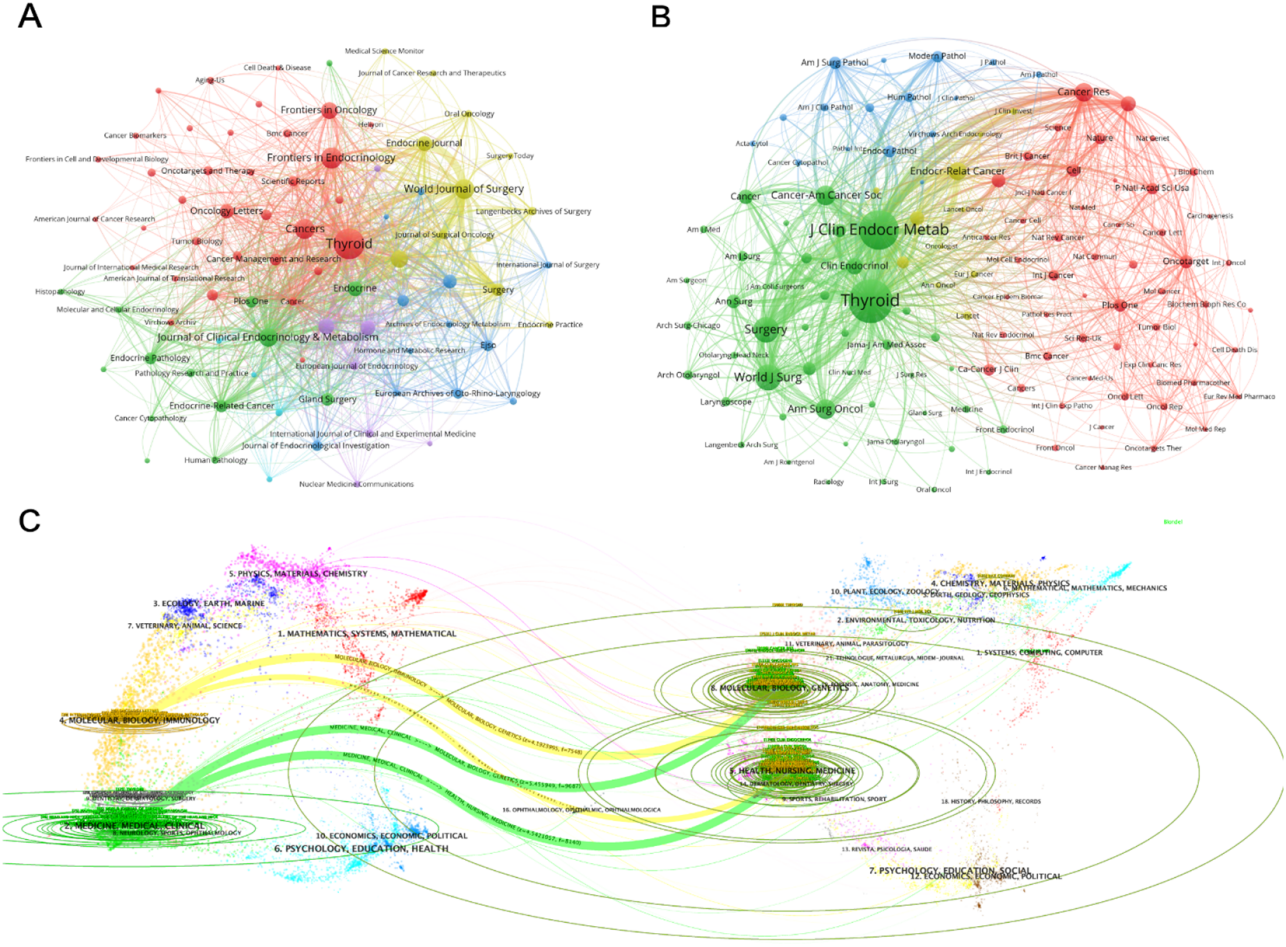
Journal citation network mapping of PTC prognosis from 2004 to 2024. **(A)** The VOSviewer visualization examines collaborative relationships among journals, with nodes representing journals publishing over ten documents and colored according to their cluster membership. Node size reflects the frequency of these journals’ presence in the network. **(B)** The VOSviewer visualization enables exploration of connections between journals, with node size indicating the frequency of citations and reflecting the significance and influence of the journals in the network. **(C)** The dual-map of PTC prognosis research journals is presented, with the left side depicting clusters of citing journals and the right side representing the cited journals. The colored trajectories between them indicate the citation relationships.

The citation and collaboration relationships among these journals are visually depicted in **Figure 6A**, offering a more intuitive understanding of their interactions. *Thyroid* prominently emerges as a core journal in the field and is closely linked to *World Journal of Surgery*, *Surgery*, and *Endocrine Journal*. **Figure 6B** visualizes the research domains represented by these journals through distinct colored clusters. For example, journals focusing on medicine and surgery, such as the *Journal of Clinical Endocrinolo*gy and Metabolism, *Surgery*, and *World Journal of Surgery*, are grouped in the green cluster. Journals covering research content in the fields of molecular biology, cell biology, and pathology, such as *Acta Cytologica*, *Cancer Cytopathology*, *Endocrine Pathology*, and *The Journal of Pathology*, are clustered in the blue category. Journals related to cancer research or endocrinology, including *Endocrine-Related Cancer*, *Oncotarget*, and *CA: A Cancer Journal for Clinicians*, are highlighted in the red or yellow clusters.

**Figure 6C** provides additional clarification on the citation and co-citation relationships among journals through an overlay analysis obtained using CiteSpace. The citing journals are listed on the right, whereas the cited journals are listed on the left. The thickness of the lines between them indicates the strength of the citation relationship. It is apparent that the citing journals primarily originate from fields such as medicine, molecular biology, immunology, and clinical studies, whereas the cited journals predominantly belong to domains such as molecular genetics, health, and medicine.

### 3.6 Keywords analysis

**Table 5** presents the top 20 keywords based on occurrence frequency and total link strength, which represent the main directions or core viewpoints of the research and can accurately reflect the research hotspots and frontiers in the field during a specific time period. The most frequently occurring keyword is papillary thyroid carcinoma (1845 occurrences), followed by thyroid cancer (524 occurrences) and prognosis (444 occurrences). Additionally, there are keywords related to the pathological manifestations and treatment targets of PTC, such as lymph node metastasis, biomarker, and BRAF V600E.

**Table 5:** Ranking of the top twenty major keywords of PTC prognosis from 2004 to 2024.

Rank	Keyword	Occurrences	Total link strength	Rank	Keyword	Occurrences	Total link strength
1	papillary thyroid carcinoma	1845	2769	11	differentiated thyroid cancer	81	159
2	thyroid cancer	524	857	12	cancer	80	161
3	prognosis	444	911	13	immunohistochemistry	77	128
4	braf	170	378	14	lymph node	68	178
5	lymph node metastasis	163	378	15	radioactive iodine	65	150
6	thyroid	156	304	16	follicular thyroid cancer	62	173
7	recurrence	155	349	17	lncrna	58	108
8	braf v600e	121	220	18	nomogram	58	122
9	metastasis	116	242	19	survival	58	143
10	biomarker	92	211	20	microrna	57	132

**Figure 7:**
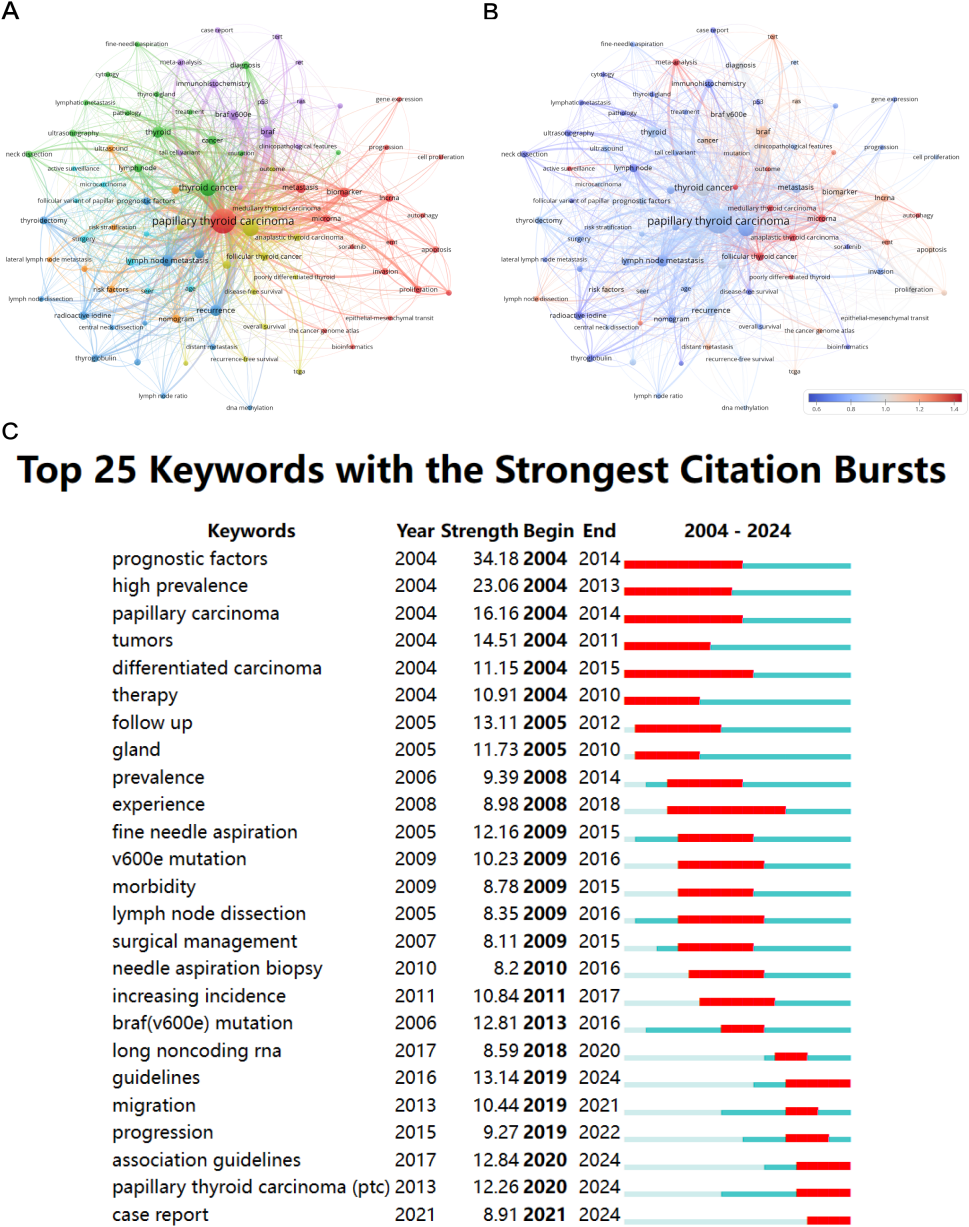
Keywords co-occurrence network mapping of PTC prognosis from 2004 to 2024. **(A)** The keyword map of PTC prognosis research visually displays the connections among studied keywords. Nodes, distinguished by various colors, represent different keyword clusters. Node size reflects co-occurrence frequency, and connections between nodes depict relationships among keywords. **(B)** The figure depicts institutions’ recent contributions to PTC prognosis research relative to their overall output from 2004 to 2024, with a red bias indicating increased influence and a blue bias suggesting decreased activity in the field. The color scale reflects the ratio of keywords over the past five years, highlighting institutions with notable impacts or reduced involvement in this study. **(C)** The diagram illustrates the 25 primary keywords characterized by pronounced bursts of citations, denoted by red spikes on the timeline. These spikes signify sudden surges in citation counts, signaling pivotal moments of emerging crucial questions or solutions within the field.

**Figures 7A, B** depict the co-occurrence relationships and strengths among key terms, not only reflecting the overlap of research content but also aiding in the identification of potential connections between research topics and paradigms. This is valuable for researchers to explore new research directions. As shown in **Figure 7A**, the key term with the highest co-occurrence strength is papillary thyroid carcinoma, which is located at the center. It forms a red cluster with some terms related to cell biology and molecular biology on the right, such as microRNA, proliferation, apoptosis, and biomarker. The yellow cluster below mainly consists of terms related to cancer, such as TCGA (The Cancer Genome Atlas), anaplastic thyroid carcinoma, follicular thyroid cancer, etc. The orange cluster mainly lists terms related to risk factors and disease progression of PTC, such as risk factors, lateral node metastasis, risk stratification, etc. The keywords in the deep blue and light blue clusters located at the bottom left are mainly related to clinical medicine and surgery, such as thyroidectomy, radioactive iodine, surgery. The keywords in the green cluster focus on describing the pathological manifestations of PTC and clinical or laboratory diagnostic methods, such as pathology, ultrasonography, fine-needle aspiration, etc. The purple cluster displays some terms related to cell biology or molecular biology related to PTC, such as immunohistochemistry, BRAF V600E, etc. **Figure 7B** includes a description of the contribution strength of keywords. It is evident that in recent years, the high-temperature keywords mainly describe nouns related to oncology, such as anaplastic thyroid carcinoma, follicular thyroid cancer, medullary thyroid carcinoma, etc.

**Figure 7C** presents the top 25 keywords exhibiting the most robust citation bursts. The keywords with the highest burst strengths are prognostic factors (34.18) and high prevalence (23.06), indicating their early emergence as research hotspots. The keyword with the longest duration of sustained research interest is differentiated carcinoma (2004-2015). Below, there are also several keywords still experiencing bursts, demonstrating that research on these topics remains current hotspots, such as guidelines (13.14), association guidelines (12.84), PTC (papillary thyroid carcinoma) (12.26), and case report (8.91).

### 3.7 Highly cited references analysis

The citation count is an important indicator for assessing the quality and impact of articles. Highly cited articles play a crucial role in driving research progress and reflecting research trends. **Table 6** presents basic information on the top fifteen highly cited articles, with the highest citation count attributed to the review by Prahallad, A et al., published in 2012 in *Nature*, titled “Unresponsiveness of colon cancer to BRAF(V600E) inhibition through feedback activation of EGFR” (22) (1499 citations). This article primarily addresses the issue of the poor efficacy of the drug PL4032 in patients with BRAF (V600E) mutant colorectal cancer. It analyzes the causes and mechanisms of action of BRAF (V600E), proposing a combination therapy using BRAF and EGFR inhibitors was proposed to treat patients with BRAF (V600E) mutant colorectal cancer. The second most highly cited literature, with 764 citations, is the article titled “BRAF mutation predicts a poorer clinical prognosis for papillary thyroid cancer” (23) authored by Xing, MZ et al. and published in the *Journal of Clinical Endocrinology and Metabolism* in 2005. Given the early publication of this article, we can infer that its research findings laid the groundwork for many subsequent studies. Through a multicenter study, this study explored the associations between BRAF mutations and the occurrence of extrathyroidal invasion, lymph node metastasis, and advanced tumor stages III/IV in patients with PTC. These findings underscore the importance of BRAF in disease diagnosis, progression, and its potential role as a therapeutic target. In the third article, also authored by Xing, MZ et al. and published in 2013 in *JAMA - Journal of the American Medical Association*, titled “Association Between BRAF V600E Mutation and Mortality in Patients With Papillary Thyroid Cancer” (24), the author further investigated the impact of BRAF on patients with PTC. Through a retrospective multicenter study, they reported an association between the BRAF (V600E) mutation and increased mortality in patients with PTC, highlighting the significant regulatory role of BRAF in the prognosis of PTC patients.

**Table 6:** Ranking of the top fifteen major highly cited references of PTC prognosis from 2004 to 2024.

Rank	Author	Article title	Source title	Cited	Year	Category	DOI
1	Prahallad, A; Sun, C; Huang, SD; Di Nicolantonio, F; Salazar, R; Zecchin, D; Beijersbergen, RL; Bardelli, A; Bernards, R	Unresponsiveness of colon cancer to BRAF(V600E) inhibition through feedback activation of EGFR	NATURE	1499	2012	Article	10.1038/nature10868
2	Xing, MZ; Westra, WH; Tufano, RP; Cohen, Y; Rosenbaum, E; Rhoden, KJ; Carson, KA; Vasko, V; Larin, A; Tallini, G; Tolaney, S; Holt, EH; Hui, P; Umbricht, CB; Basaria, S; Ewertz, M; Tufaro, AP; Califano, JA; Ringel, MD; Zeiger, MA; Sidransky, D; Ladenson, PW	BRAF mutation predicts a poorer clinical prognosis for papillary thyroid cancer	JOURNAL OF CLINICAL ENDOCRINOLOGY & METABOLISM	764	2005	Article	10.1210/jc.2005-0987
3	Xing, MZ; Alzahrani, AS; Carson, KA; Viola, D; Elisei, R; Bendlova, B; Yip, L; Mian, C; Vianello, F; Tuttle, RM; Robenshtok, E; Fagin, JA; Puxeddu, E; Fugazzola, L; Czarniecka, A; Jarzab, B; O’Neill, CJ; Sywak, MS; Lam, AK; Riesco-Eizaguirre, G; Santisteban, P; Nakayama, H; Tufano, RP; Pai, SI; Zeiger, MA; Westra, WH; Clark, DP; Clifton-Bligh, R; Sidransky, D; Ladenson, PW; Sykorova, V	Association Between BRAF V600E Mutation and Mortality in Patients With Papillary Thyroid Cancer	JAMA-JOURNAL OF THE AMERICAN MEDICAL ASSOCIATION	701	2013	Article	10.1001/jama.2013.3190
4	Ito, Y; Miyauchi, A; Kihara, M; Higashiyama, T; Kobayashi, K; Miya, A	Patient Age Is Significantly Related to the Progression of Papillary Microcarcinoma of the Thyroid Under Observation	THYROID	555	2014	Article	10.1089/thy.2013.0367
5	Lundgren, CI; Hall, P; Dickman, PW; Zedenius, J	Clinically significant prognostic factors for differentiated thyroid carcinoma - A population-based, nested case-control study	CANCER	545	2006	Article	10.1002/cncr.21653
6	Randolph, GW; Duh, QY; Heller, KS; LiVolsi, VA; Mandel, SJ; Steward, DL; Tufano, RP; Tuttle, RM	The Prognostic Significance of Nodal Metastases from Papillary Thyroid Carcinoma Can Be Stratified Based on the Size and Number of Metastatic Lymph Nodes, as Well as the Presence of Extranodal Extension	THYROID	540	2012	Article	10.1089/thy.2012.0043
7	Xing, MZ; Liu, RY; Liu, XL; Murugan, AK; Zhu, GW; Zeiger, MA; Pai, S; Bishop, J	BRAF V600E and TERT Promoter Mutations Cooperatively Identify the Most Aggressive Papillary Thyroid Cancer With Highest Recurrence	JOURNAL OF CLINICAL ONCOLOGY	505	2014	Article	10.1200/JCO.2014.55.5094
8	Leboulleux, S; Rubino, C; Baudin, E; Caillou, B; Hartl, DM; Bidart, JM; Travagli, JP; Schlumberger, M	Prognostic factors for persistent or recurrent disease of papillary thyroid carcinoma with neck lymph node metastases and/or tumor extension beyond the thyroid capsule at initial diagnosis	JOURNAL OF CLINICAL ENDOCRINOLOGY & METABOLISM	448	2005	Article	10.1210/jc.2005-0285
9	Ito, Y; Higashiyama, T; Takamura, Y; Kobayashi, K; Miya, A; Miyauchi, A	Clinical Outcomes of Patients with Papillary Thyroid Carcinoma after the Detection of Distant Recurrence	WORLD JOURNAL OF SURGERY	417	2010	Article	10.1007/s00268-010-0712-0
10	Xing, MZ; Alzahrani, AS; Carson, KA; Shong, YK; Kim, TY; Viola, D; Elisei, R; Bendlová, B; Yip, L; Mian, C; Vianello, F; Tuttle, RM; Robenshtok, E; Fagin, JA; Puxeddu, E; Fugazzola, L; Czarniecka, A; Jarzab, B; O’Neill, CJ; Sywak, MS; Lam, AK; Riesco-Eizaguirre, G; Santisteban, P; Nakayama, H; Clifton-Bligh, R; Tallini, G; Holt, EH; Sykorová, V	Association Between BRAF V600E Mutation and Recurrence of Papillary Thyroid Cancer	JOURNAL OF CLINICAL ONCOLOGY	404	2015	Article	10.1200/JCO.2014.56.8253
11	Elisei, R; Ugolini, C; Viola, D; Lupi, C; Biagini, A; Giannini, R; Romei, C; Miccoli, P; Pinchera, A; Basolo, F	BRAFV600E mutation and outcome of patients with papillary thyroid carcinoma:: A 15-year median follow-up study	JOURNAL OF CLINICAL ENDOCRINOLOGY & METABOLISM	404	2008	Article	10.1210/jc.2008-0607
12	Capper, D; Preusser, M; Habel, A; Sahm, F; Ackermann, U; Schindler, G; Pusch, S; Mechtersheimer, G; Zentgraf, H; von Deimling, A	Assessment of BRAF V600E mutation status by immunohistochemistry with a mutation-specific monoclonal antibody	ACTA NEUROPATHOLOGICA	383	2011	Article	10.1007/s00401-011-0841-z
13	Adeniran, AJ; Zhu, ZW; Gandhi, M; Steward, DL; Fidler, JP; Giordano, TJ; Biddinger, PW; Nikiforov, YE	Correlation between genetic alterations and microscopic features, clinical manifestations, and prognostic characteristics of thyroid papillary carcinomas	AMERICAN JOURNAL OF SURGICAL PATHOLOGY	380	2006	Article	10.1097/01.pas.0000176432.73455.1b
14	Rahbari, R; Zhang, LS; Kebebew, E	Thyroid cancer gender disparity	FUTURE ONCOLOGY	326	2010	Review	10.2217/FON.10.127
15	Lupi, C; Giannini, R; Ugolini, C; Proietti, A; Berti, P; Minuto, M; Materazzi, G; Elisei, R; Santoro, M; Miccoli, P; Basolo, F	Extensive clinical experience - Association of BRAF V600E mutation with poor clinicopathological outcomes in 500 consecutive cases of papillary thyroid carcinoma	JOURNAL OF CLINICAL ENDOCRINOLOGY & METABOLISM	326	2007	Article	10.1210/jc.2007-1179

**Figure 8:**
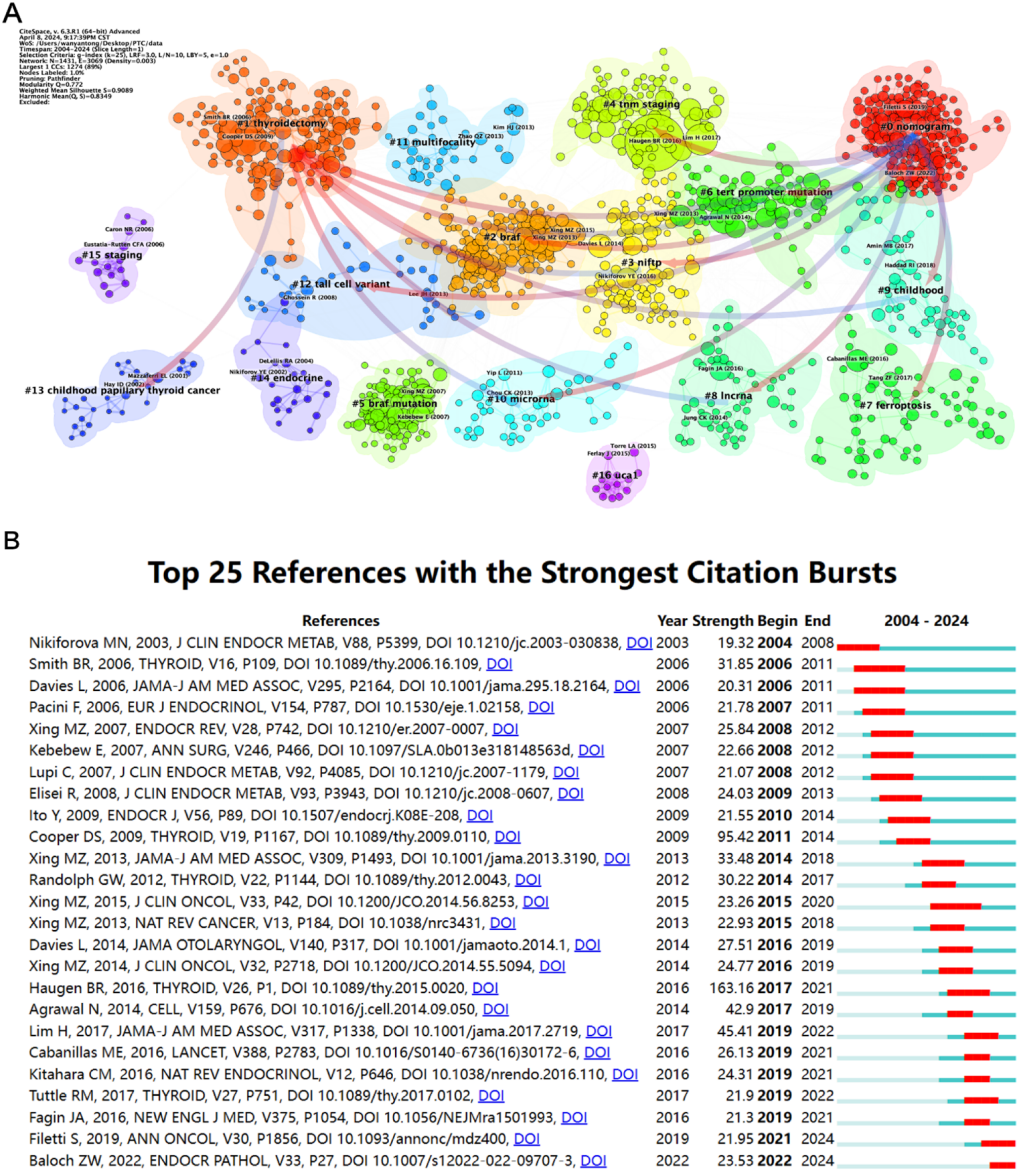
Highly cited references co-citation network mapping of PTC prognosis from 2004 to 2024. **(A)** The keyword heatmap displays topics, with smaller numbers indicating larger clusters, and #0 representing the largest cluster. Node size reflects co-citation frequency, and the links between nodes signify co-citation relationships. **(B)** The diagram illustrates the 25 primary references characterized by pronounced bursts of citations, denoted by red spikes on the timeline. These spikes signify sudden surges in citation counts, signaling pivotal moments of emerging crucial questions or solutions within the field.

**Figure 8A** illustrates the co-citation patterns of the literature related to PTC prognosis. By connecting clusters, it delineates the developmental and evolving sequence of core concepts within these highly cited publications. The largest cluster, labeled as #0 nomogram, is noteworthy as it is the latest-formed cluster in the graph and its connections extend to multiple clusters, indicating that research on PTC prognosis from this perspective has emerged and evolved as an important research direction since the early 21st century, integrating and developing various research contents over time. Key concepts influencing this cluster include #4 TNM (TNM Classification of Malignant Tumors), #3 NIFTP (Noninvasive follicular thyroid neoplasm with papillary), #2 BRAF, and #1 thyroidectomy. In terms of the overall developmental trend of research content, the early concept #15 staging gradually materializes into the more mature #4 TNM. Research on PTC has evolved from # childhood PTC and early surgical treatment methods # thyroidectomy to delving deeper into the disease mechanisms, mainly based on #2 BRAF as a therapeutic target for developing novel intervention strategies.

**Figure 8B** illustrates the reference with the highest burst intensity, which is the work by Haugen BR, published in 2016. Titled “2015 American Thyroid Association Management Guidelines for Adult Patients with Thyroid Nodules and Differentiated Thyroid Cancer: The American Thyroid Association Guidelines Task Force on Thyroid Nodules and Differentiated Thyroid Cancer” (25), it has a burst intensity of 163.16. One year after publication, this reference gained widespread attention and continues to attract significant interest (2017–2021). Cooper DS’s work, published in 2009 and titled “Revised American Thyroid Association Management Guidelines for Patients with Thyroid Nodules and Differentiated Thyroid Cancer: The American Thyroid Association (ATA) Guidelines Taskforce on Thyroid Nodules and Differentiated Thyroid Cancer” (8), follows closely with a burst intensity of 95.42. Furthermore, the relatively recent references “Thyroid cancer: ESMO Clinical Practice Guidelines for diagnosis, treatment and follow-up” (9) by Filetti S et al. and “Overview of the 2022 WHO Classification of Thyroid Neoplasms” (26) by Baloch ZW et al., are still experiencing bursts, indicating the ongoing interest and impact of these articles.

## 4 Discussion

In this study, we conducted a bibliometric analysis of 3,430 articles related to the prognosis of PTC sourced from the Web of Science Core Collection database. Over the past two decades, we have observed a yearly increase in the number of studies focused on PTC prognosis. This trend indicates a growing recognition among international scholars of the importance of evaluating the prognosis of PTC, rather than solely focusing on treatment. In 2022, the number of publications and citations on PTC prognosis reached an all-time high. However, our analysis of publication and citation trends revealed periods of plateau in the number of articles and citations related to PTC prognosis in recent years. One possible explanation for these plateaus could be the influence of key issues within the academic community, such as the debate surrounding thyroid cancer overdiagnosis and overtreatment. Over the past few years, the potential for overdiagnosis of thyroid cancer has been increasingly recognized (27, 28). This might lead to a reevaluation of treatment strategies, with a shift towards more conservative approaches in certain cases. Such shifts in clinical practice and research focus can result in a temporary slowdown in publication activity as researchers and clinicians reassess existing knowledge and practices. Understanding these dynamics is crucial for interpreting the trends observed in our study and highlights the importance of aligning research efforts with evolving clinical needs and priorities. Bibliometric research specifically targeting PTC prognosis remains limited, highlighting the need for our supplementary study. Through our analysis, we aimed to assist clinicians in precisely understanding current research hotspots and trends in PTC prognosis, thereby improving patient quality of life and the quality of medical services.

China and the United States lead in both the volume of publications and the number of citations in this field. Although China has a higher total publication volume, the citation rate is slightly lower than that of the United States, likely due to differences in the duration and international recognition of research in this area between the two countries. Other countries, such as South Korea, Italy, and Japan, have also made significant contributions. We generated a chord diagram to illustrate the collaboration intensity among different countries in this field. It is evident that the United States collaborates closely with China and Italy, and South Korea plays a positive role in collaboration, particularly with the United States. This suggests that leading countries in this field tend to have stronger collaborations with each other, whereas cooperation with other countries is less frequent. Compared with developed countries, most developing countries have less research in this area. Similar patterns are observed among authors, with top-ranking authors primarily from South Korea, Japan, the United States, and China, and most collaborations occurring within the same country and institution. Therefore, there is a need to enhance international collaboration in this field to bridge the research gap among countries.

As issues related to PTC prognosis continue to emerge, various institutions from different countries have gradually begun related research. We ranked institutions based on the number of publications and total citations in this field. Our research revealed that Japan’s Kuma Hospital, South Korea’s Yonsei University, and China’s Shanghai Jiao Tong University are the top three institutions in terms of publication volume, indicating a high level of attention and more comprehensive clinical data and in-depth research in PTC prognosis. Among the top ten institutions in terms of publication volume, half are from China, demonstrating China’s significant contribution to this field. Additionally, we visualized the collaborative relationships among institutions. Spatially, institutions from the United States and European countries exhibit much tighter collaboration, far exceeding their cooperation with institutions from other regions. China and South Korea primarily collaborate domestically, with large clusters of institutions enhancing the closeness of domestic cooperation. From a temporal perspective, after foundational work by institutions in Europe, the United States, Japan, and South Korea, Chinese institutions have emerged as new contributors to the field of PTC prognosis in recent years, providing more comprehensive and refined content. With respect to institutional contributions, our findings indicate that certain institutions, such as Kuma Hospital in Japan, have been particularly prolific in publishing research on PTC prognosis. This concentration of research output may be attributed to several factors. First, Kuma Hospital has a well-established research infrastructure and dedicated resources for thyroid cancer research, allowing for sustained and focused investigations in this area (29). Additionally, the institution’s collaborative network with other leading research centers facilitates the sharing of expertise and resources, further enhancing its research output (30). This clustering of research efforts at a single institution can lead to a deeper exploration of specific topics, such as active surveillance, and contribute significantly to the overall body of knowledge concerning PTC prognosis. However, it also highlights the need for broader international collaboration to ensure diverse perspectives and approaches in future research.

Journal analysis shows that *Thyroid*, *Frontiers in Endocrinology*, and the *World Journal of Surgery* are leading in publication volume, while Thyroid, the Journal of Clinical Endocrinology & Metabolism, and the World Journal of Surgery lead in co-citation frequency, indicating strong competitiveness in this field. As shown in **Table 4**, Thyroid consistently ranks first across various metrics, reflecting its broad research and innovative ideas in PTC prognosis. **Figure 6** illustrates the different research areas wo which these journals belong, such as the *Journal of Clinical Endocrinology & Metabolism*, *Surgery*, and *Journal of Surgery*, which are related to medicine and surgery. Meanwhile, *Acta Cytologica*, *Tumor Biology*, *Endocrine Pathology*, and *Journal of Pathology* are categorized under molecular biology, cell biology, and pathology. Journals such as the *Endocrine-Related Cancer*, *Oncotarget*, and *CA: A Cancer Journal for Clinicians* belong to cancer research or endocrinology, which is consistent with the dual-map overlay analysis in **Figure 6C**. Notably, combined with our chart analysis, we found that most publications on PTC prognosis are published in journals related to clinical research and surgery, indicating substantial research potential in basic fields.

Through our analysis of clinical studies and literature reports on PTC prognosis over the past two decades, we identified the top 20 keywords by frequency and total link strength. In addition to “papillary thyroid carcinoma,” “thyroid cancer,” and “prognosis,” other keywords related to the pathological features and therapeutic targets of PTC have emerged. High-frequency keywords and co-occurrence cluster analysis revealed current hotspots and major research directions. As shown in **Figures 7A**, these keywords are broadly divided into six clusters. The keyword with the highest co-occurrence strength, “papillary thyroid carcinoma,” is at the center. Terms related to cell and molecular biology, such as “microRNA,” “proliferation,” “apoptosis,” and “biomarkers,” are in the red cluster on the right side of the figure. Liviu Hitu et al. revealed the regulatory patterns of microRNAs in PTC cells, demonstrating that most microRNAs regularly expressed in normal thyroids have significant tumor-suppressive effects in PTC (31). Xu Yuan et al. reported that si-STAT3 could inhibit p-STAT3 protein expression, reduce cell proliferation and metastasis, and induce apoptosis in thyroid carcinoma following miRNA-148a overexpression (32). These studies highlight the importance of molecular biology in PTC treatment and prognosis.

The cancer-related terms like “The Cancer Genome Atlas (TCGA),” “anaplastic thyroid cancer,” and “follicular thyroid cancer” are in the yellow cluster at the bottom. TCGA is a large dataset containing clinical data from various human cancers and is an essential resource for cancer researchers. For PTC research, the TCGA is indispensable for understanding the disease mechanisms. Wang Yinghao’s team used RNA-seq data from the TCGA database to identify that UNC5B-AS1 is an oncogene that regulates PTC cell proliferation, migration, and invasion (33). By comparing PTC-related cancer data and database analyses, we gained a more comprehensive understanding of the clinical diagnosis, treatment, and prognosis of PTC.

Terms associated with PTC risk factors and disease progression, such as “risk factors,” “lateral lymph node metastasis,” and “risk stratification,” are in the orange cluster. Zhao Hengqiang et al. analyzed clinical data from 721 thyroid cancer patients to identify risk factors for skip metastasis and lateral lymph node metastasis in PTC, and concluded that comprehensive central compartment dissection could reduce skip metastasis (34). Investigating potential risks in clinical cases to predict and mitigate postoperative complications and recurrence is a current hotspot in PTC prognosis research.

The green and blue clusters contain keywords related to clinical treatment. The blue cluster includes “thyroidectomy” and “radioactive iodine,” whereas the green cluster includes “pathology,” “ultrasound,” and “fine-needle aspiration,” which are common diagnostic and treatment methods in clinical practice. Accurate diagnosis and targeted surgical treatment are crucial for improving PTC prognosis. Thus, related clinical research is highly favored. Keywords such as “immunohistochemistry” and “BRAF V600E” are in the purple cluster. From a temporal perspective, early research hotspots focused on prognostic factors and high prevalence. In recent years, case reports and association guidelines have gained prominence.

Highly cited articles usually have significant academic influence and indicate research quality. The most cited articles include “Unresponsiveness of colon cancer to BRAF(V600E) inhibition through feedback activation of EGFR,” “BRAF mutation predicts a poorer clinical prognosis for papillary thyroid cancer,” and “Association Between BRAF V600E Mutation and Mortality in Patients With Papillary Thyroid Cancer.” All three studies involved BRAF V600E, highlighting its critical role in PTC pathogenesis and early detection. Research has shown that BRAF mutations are closely related to aggressive clinical features and poor prognosis in thyroid cancer patients (35).

The co-citation analysis of PTC prognosis literature revealed the evolution and integration of research outcomes over time. A high citation frequency indicates that researchers’ understanding of PTC prognosis has progressed from basic concepts and methods to more mature perspectives and techniques. Finally, the analysis of burst intensity in related literature shows that most top-ranked documents are guidelines, likely due to their authoritative nature and reliable data sources, leading to sustained interest and citation.

There are several inherent limitations to this approach. First, we selected the Web of Science Core Collection (WOSCC) database to increase the quality of studies. This database is considered comprehensive and authoritative in the medical field, offering more accurate document type categorization than other databases (36). Nevertheless, the quality of the articles included in WOSCC can vary, which may introduce some bias into our findings. Second, some newly published articles that might be highly significant were not included because of the lag in publication time and were therefore not captured within our analysis period. Third, our search strategy relied on the Topic Search (TS) field, which restricts the search to titles, abstracts, and author keywords. Consequently, this approach may inadvertently exclude relevant studies where prognostic indicators are discussed extensively in the full text but are not explicitly captured in the abstract or keywords. Future systematic reviews could consider combining title/abstract/keyword searches with full-text retrieval to address this limitation. Fourth, the literature search was conducted on April 9, 2024. Therefore, studies published after this cutoff date were not included in the analysis. Given the rapid pace of research in oncology and molecular biology, the findings may not reflect the most recent developments in the field. Finally, this study included only articles written in English, potentially overlooking relevant literature in other languages.

## 5 Conclusion

Through bibliometric analysis, the prognosis of PTC has emerged as a core research area. As PTC constitutes the majority of thyroid cancers, it is natural that most studies are related to PTC. Our analysis identified several important points that warrant further attention. First, the steady increase in research volume and the emergence of key clusters in areas such as molecular biology, risk factors, and clinical management highlight the growing complexity and multidisciplinary nature of PTC prognosis research. Second, the prominent role of certain institutions and journals underscores the importance of collaborative efforts in advancing knowledge in this field. Additionally, the high citation rates of articles focusing on BRAF V600E mutations suggest that genetic factors continue to be a critical area of investigation. These insights from our bibliometric analysis provide valuable directions for future research, emphasizing the need for continued exploration of genetic markers, risk stratification, and personalized treatment strategies to improve patient outcomes and the quality of prognostic care for PTC patients.

## Data Availability

The original contributions presented in the study are included in the article/supplementary material. Further inquiries can be directed to the corresponding author.
